# Difference in patterns of retinal ganglion cell damage between primary open-angle glaucoma and non-arteritic anterior ischaemic optic neuropathy

**DOI:** 10.1371/journal.pone.0187093

**Published:** 2017-10-26

**Authors:** Yeon Hee Lee, Kyoung Nam Kim, Dong Won Heo, Tae Seen Kang, Sung Bok Lee, Chang-sik Kim

**Affiliations:** Department of Ophthalmology, Chungnam National University Hospital, Chungnam National University School of Medicine, Daejeon, Korea; University of Florida, UNITED STATES

## Abstract

**Purpose:**

To compare the patterns of retinal ganglion cell damage between primary open-angle glaucoma (POAG) and non-arteritic anterior ischaemic optic neuropathy (NAION).

**Methods:**

In total, 35 eyes with unilateral NAION, and 70 age- and average peripapillary retinal nerve fibre layer (RNFL) thickness-matched eyes with POAG, were enrolled as disease groups; 35 unaffected fellow eyes of the NAION, and 70 age- and refractive error-matched normal subjects for the POAG, were enrolled as their control groups, respectively. The peripapillary RNFL thickness and macular ganglion cell plus inner plexiform layer (GCIPL) thickness were compared between the disease groups and their controls, and between the two disease groups.

**Results:**

Mean RNFL thicknesses at the 1 and 2 o’clock (superonasal) positions were thinner in NAION than in POAG (both *p* < 0.05). Mean RNFL thickness at 7 o’clock (inferotemporal) was thinner in POAG than in NAION (*p* = 0.001). Although there was no significant difference between NAION and POAG in average GCIPL thickness, all of the sectoral GCIPL thicknesses were thinner in NAION (all *p* < 0.05), except in the inferior and inferotemporal sectors. The ranges of the clock-hour RNFL with damage greater than the average RNFL thickness reduction, versus fellow eyes and control eyes, were 7 hours in NAION and 4 hours in POAG.

**Conclusions:**

The more damaged clock-hour RNFL regions differed between NAION (1 and 2 o’clock) and POAG (7 o’clock). Most sectoral GCIPL thicknesses were thinner in NAION than in POAG.

## Introduction

Glaucoma, the most common type of progressive optic neuropathy, is characterised by primary axonal loss and subsequent retinal ganglion cell loss. [1, 2] Management of glaucoma has high costs and extends over a long time period; it may even be life-long. Also, the management itself can substantially reduce the patient’s quality of life. [3–5] Another common optic neuropathy in the elderly, non-arteritic anterior ischaemic optic neuropathy (NAION), is caused by insufficient perfusion to the posterior ciliary arteries, which supply blood to the optic nerve head. [6–8] Similar to glaucoma, NAION also results in significant axonal and ganglion cell loss. [9–13] However, unlike glaucoma, NAION does not progress chronologically and to date there is no effective treatment strategy, so there is no need for exhaustive care (except management of vascular risk factors to prevent NAION occurring in the fellow eye). [14] Consequently, in patients with NAION, it is important to prevent unnecessary overtreatment that could result from misdiagnosis of NAION as glaucoma.

Traditionally, optic disc pallor without concomitant enlargement or excavation of optic disc cupping is considered a pointer in the differential diagnosis of NAION versus glaucoma. [15] However, occasional cases of NAION with visual field loss and optic disc cupping with or without intraocular pressure elevation may be misdiagnosed as primary open-angle glaucoma. [16–19] There is a difference in the mechanism producing altitudinal asymmetry and optic disc damage in the two diseases. In glaucoma, altitudinal asymmetry is associated with a characteristic distribution of retinal nerve fibres. The temporal retinal nerve fibres originate on either side of a horizontal raphe and form an arch above or below the fovea; arcuate nerve fibres occupy the superotemporal and inferotemporal portions of the optic nerve head. [20, 21] In contrast, in patients with NAION, altitudinal asymmetry is associated with a characteristic distribution of blood vessels consisting of distinctive upper and lower halves within the vascular circle, which supplies the optic disc. [22–24] We anticipated that the patterns of retinal ganglion cell damage resulting from the two diseases would differ. Ocular imaging techniques that include optical coherence tomography (OCT) are used to measure changes and structural damage resulting from glaucoma. [25–27] Spectral-domain OCT can measure the thickness of both the macular ganglion cell plus inner plexiform layer (GCIPL) and the peripapillary retinal nerve fibre layer (RNFL) to assess the ganglion cell bodies/dendrites and their axons, respectively. [25, 28]

In this cross-sectional study using spectral-domain OCT (Cirrus; Carl Zeiss Meditec, Dublin, CA, USA), we analysed both sectoral RNFL and sectoral GCIPL in primary open-angle glaucoma (POAG) patients and stabilised, unilateral NAION patients. Similar to our study, Fard et al. [13] compared patterns of GCIPL defects between POAG and NAION using another spectral-domain OCT device (Spectralis; Heidelberg, Jena, Germany). In their study, to match the severity of optic disc damage in NAION, moderate-to-severe POAG cases (mean deviation [MD] on visual field examination < -6.0 dB) were selectively enrolled. However, very low visual acuity is not unusual in NAION and is a known limitation on reliable visual field examinations; the mean visual acuity of the NAION patients in their study was 0.92 logMAR. [5, 29] Thus, we used average RNFL thickness, instead of MD, as a more objective and reliable matching index in the diseased groups. [30, 31]

## Methods

This retrospective, cross-sectional study was approved by the Institutional Review Board (IRB) of Chungnam National University Hospital, which waived the requirement for informed consent from participants. It was conducted in accordance with all relevant requirements of the Declaration of Helsinki. Patients with unilateral NAION were consecutively enrolled at our Department of Ophthalmology from May 1, 2013, to April 31, 2016. During the same period, POAG patients matched to the NAION group by age, gender, and average RNFL thickness, and healthy subjects matched to the POAG group by age, gender, and refractive error, were enrolled. All patients underwent a thorough eye examination, including measurement of best-corrected visual acuity (BCVA), auto-refractometry, slit-lamp biomicroscopy, Goldmann applanation tonometry, dilated fundus examination, and Cirrus HD OCT (Carl Zeiss Meditec).

### Unilateral NAION and fellow eyes

The NAION diagnosis was based on sudden, painless loss of visual acuity and optic disc oedema, regardless of superficial disc haemorrhages in the acute phase. Arteritic anterior ischaemic optic neuropathy was ruled out by the absence of systemic symptoms of giant arteritis, a high erythrocyte sedimentation rate (> 50 mm/h), and a positive C-reactive protein. At the time of study recruitment, patients satisfying all of the following criteria were enrolled: at least 3 months had elapsed since the acute phase, optic disc swelling had subsided and disc borders were clearly demarcated, no other ocular or neurological disease apart from NAION was evident, and the fellow eye was healthy and without any pathology. Patients’ healthy fellow eyes were used as controls.

### POAG and their controls

POAG patients, matched with NAION patients in terms of age (within ±1 year), gender, and average RNFL thickness (within ±5 μm) in the affected eye, were consecutively enrolled at a ratio of 2:1. To meet the criteria for diagnosis of POAG, patients had to exhibit glaucomatous optic disc change, a reproducible glaucomatous visual field defect on the Swedish interactive threshold algorithm standard of 24–2 perimetry (Humphrey Field Analyzer II; Carl Zeiss Meditec), and open angles on gonioscopy. Glaucomatous optic disc changes were characterised as focal or diffuse neuroretinal rim thinning, localised notching, or nerve fibre layer defects with correlating visual field changes. Glaucomatous visual field defects were defined by two of the following three criteria: the presence of a cluster of three points on a pattern deviation probability plot at P < 0.05, one of which was at P< 0.01, a pattern standard deviation at P < 0.05, or glaucoma hemifield test results outside normal limits.

As controls, age-, gender-, and refractive error (spherical equivalent, within ±0.5 dioptre)-matched healthy subjects were recruited from among those who came for a routine eye examination. They had no evidence of glaucomatous optic neuropathy, defined as increased cupping (cup-to-disc ratio > 0.6), cup/disc asymmetry of > 0.2 between the study and fellow eye, rim thinning, notching, or excavation. They also denied a family history of glaucoma. In both POAG and healthy controls, we excluded subjects with high myopia (< -6.0 D), and ocular or neurological diseases other than POAG. Additionally, patients who had undergone intraocular surgery, other than uncomplicated cataract surgery performed more than 6 months earlier, were excluded. When both eyes fulfilled the inclusion criteria, one eye was randomly selected for inclusion in the study.

### Spectral-domain OCT measurements

Optic disc scans (optic disc cube 200 × 200 protocol) and macular scans (macular cube 512 × 128 protocol) using Cirrus HD OCT (Carl Zeiss Meditec), for RNFL and GCIPL thickness measurements, respectively, were performed by an experienced examiner. For inclusion, OCT images had to have a signal strength ≥ 6 and the absence of artefacts caused by eye motion, blinking, poor centration, or segmentation error. The evaluated RNFL thickness parameters were average thickness (360° measure), four-quadrant thickness (temporal, superior, nasal, inferior quadrant), and thickness at each of the 12 clock-hour positions: 12 o’clock (superior), 3 o’clock (nasal), 6 o’clock (inferior), and 9 o’clock (temporal). The analysed GCIPL thickness parameters were average and six-sectoral thicknesses (superotemporal, superior, superonasal, inferonasal, inferior, inferotemporal) in a macular elliptical annulus. [32, 33]

### Statistical analysis

Comparisons of demographics between NAION and fellow eyes were performed using linear mixed models to account for correlations between eyes; comparisons of demographics between NAION and POAG, and between POAG and healthy controls, were performed using independent t-tests. Comparisons of all RNFL and GCIPL thickness parameters between NAION eyes and fellow eyes were performed using linear mixed models with axial length as a covariate. Comparisons of all RNFL and GCIPL thickness parameters between NAION and POAG patients, and between POAG patients and healthy controls, were performed using analysis of covariance (ANCOVA) with adjustment for axial length. The Bonferroni correction was applied when multiple comparisons were performed. All statistical analyses were performed using SPSS software (ver. 18.0; SPSS, Inc., Chicago, IL, USA). P values < 0.05 were considered to indicate statistical significance.

## Results

The study included 35 stabilised unilateral NAION eyes and their 35 fellow eyes, 70 eyes of the 70 POAG patients, and 70 eyes of the 70 healthy controls. Demographic characteristics of the subjects are summarised in Table 1. Between NAION and POAG, there was no significant difference in mean age or mean axial length (63.6 ± 8.6 vs. 64.3 ± 9.2 years, *p* = 0.751; 22.5 ± 1.0 and 23.3 ± 0.9 mm, p = 0.112, respectively), but there were significant differences in mean BCVA and mean refractive error (0.5 ± 0.7 and 0.1 ± 0.2, *p* < 0.001; -0.2 ± 1.3 and -0.6 ± 1.8 dioptre, *p* = 0.024, respectively). Between NAION and their fellow eyes, only mean BCVA was noticeably worse in NAION (p < 0.001). Between POAG and their normal controls, no demographic variable was significantly different.

**Table 1:** Demographics of the subjects.

	NAION	Fellow eyes	POAG	Normal controls	*p* value		
					NAION vs. Fellow eyes*	NAION vs. POAG†	POAG vs. Normal controls†
Number of eyes	35	35	70	70			
Age (years)	63.6 ± 8.6	63.6 ± 8.6	64.4 ± 9.0	64.3 ± 9.2		0.751	0.802
Male sex (%)	42.9	42.9	42.9	42.9			
BCVA (logMAR)	0.5 ± 0.7	0.1 ± 0.2	0.1 ± 0.2	0.1 ± 0.2	<0.001	<0.001	0.623
Spherical equivalent (dioptre)	-0.2 ± 1.3	0.1 ± 1.2	-0.6 ± 1.8	-0.5 ± 1.6	0.182	0.024	0.680
Axial length (mm)	22.5 ± 1.0	22.6 ± 1.0	23.3 ± 0.9	23.3 ±0.8	0.945	0.112	0.761
Humphrey visual field test							
MD (dB)	-17.7 ± 8.7	-1.1 ± 2.5	-13.5 ± 7.5	-1.2 ± 2.3	<0.001	0.118	<0.001
PSD (dB)	7.5 ± 3.8	2.2 ± 1.4	7.6 ± 3.8	1.7 ± 1.8	<0.001	0.757	<0.001

NAION = non-arteritic anterior ischaemic optic neuropathy, POAG = primary open-angle glaucoma

BCVA = best corrected visual acuity, MD = mean deviation, PSD = pattern standard deviation

* Linear mixed model

† Independent-t test

### Comparison between NAION and POAG

Table 2 shows comparisons of the axial length adjusted RNFL thickness parameters between NAION and POAG. Average RNFL thicknesses of the NAION and POAG groups were 65.0 ± 12.3 and 64.5 ± 11.6 μm, respectively (p = 0.742). There was no difference in quadrant RNFL thickness parameters. Among the clock-hour RNFL thickness parameters, RNFL thicknesses at 1 and 2 o’clock in NAION were significantly thinner than in POAG (62.8 ± 17.3 vs. 76.1 ± 21.7 μm, *p* < 0.001, and 58.0 ± 14.8 vs. 68.2 ± 15.8 μm, *p* = 0.005, respectively). The RNFL thickness at 7 o’clock in POAG was significantly thinner than in NAION (72.0 ± 25.3 vs. 94.0 ± 29.7 μm, *p* = 0.001).

**Table 2:** Comparison of the peripapillary retinal nerve fibre layer thickness parameters between non-arteritic anterior ischaemic optic neuropathy and primary open-angle glaucoma.

	NAION	POAG	*P* value*
Average RNFL thickness	65.0 ± 12.3	64.5 ± 11.6	0.742
Quadrant RNFL thickness			
Superior	71.2 ± 22.7	76.8 ± 18.9	0.245
Nasal	54.6 ± 10.0	57.6 ± 8.7	0.203
Inferior	80.3 ± 23.6	71.2 ± 21.8	0.176
Temporal	53.4 ± 12.1	52.6 ± 13.3	0.901
Clock-hour RNFL thickness			
11 o’clock	73.9 ± 25.3	75.0 ± 23.1	0.822
12 o’clock	76.2 ± 27.4	79.3 ± 22.0	0.393
1 o’clock	62.8 ± 17.3	76.1 ± 21.7	<0.001
2 o’clock	58.0 ± 14.8	68.2 ± 15.8	0.005
3 o’clock	52.2 ± 9.5	51.8 ± 9.9	0.862
4 o’clock	53.5 ± 7.9	53.1 ± 9.1	0.729
5 o’clock	64.8 ± 16.0	66.8 ± 14.0	0.329
6 o’clock	82.2 ± 28.9	74.8 ± 21.2	0.400
7 o’clock	94.0 ± 29.7	72.0 ± 25.3	0.001
8 o’clock	55.5 ± 14.3	52.5 ± 13.2	0.407
9 o’clock	49.2 ± 10.8	49.0 ± 12.7	0.916
10 o’clock	55.4 ± 17.7	56.3 ± 17.5	0.732

(μm, mean ± SD)

NAION = non-arteritic anterior ischaemic optic neuropathy, POAG = primary open-angle glaucoma

RNFL = retinal nerve fibre layer

* Axial length adjusted analysis of covariance.

**Fig 1:**
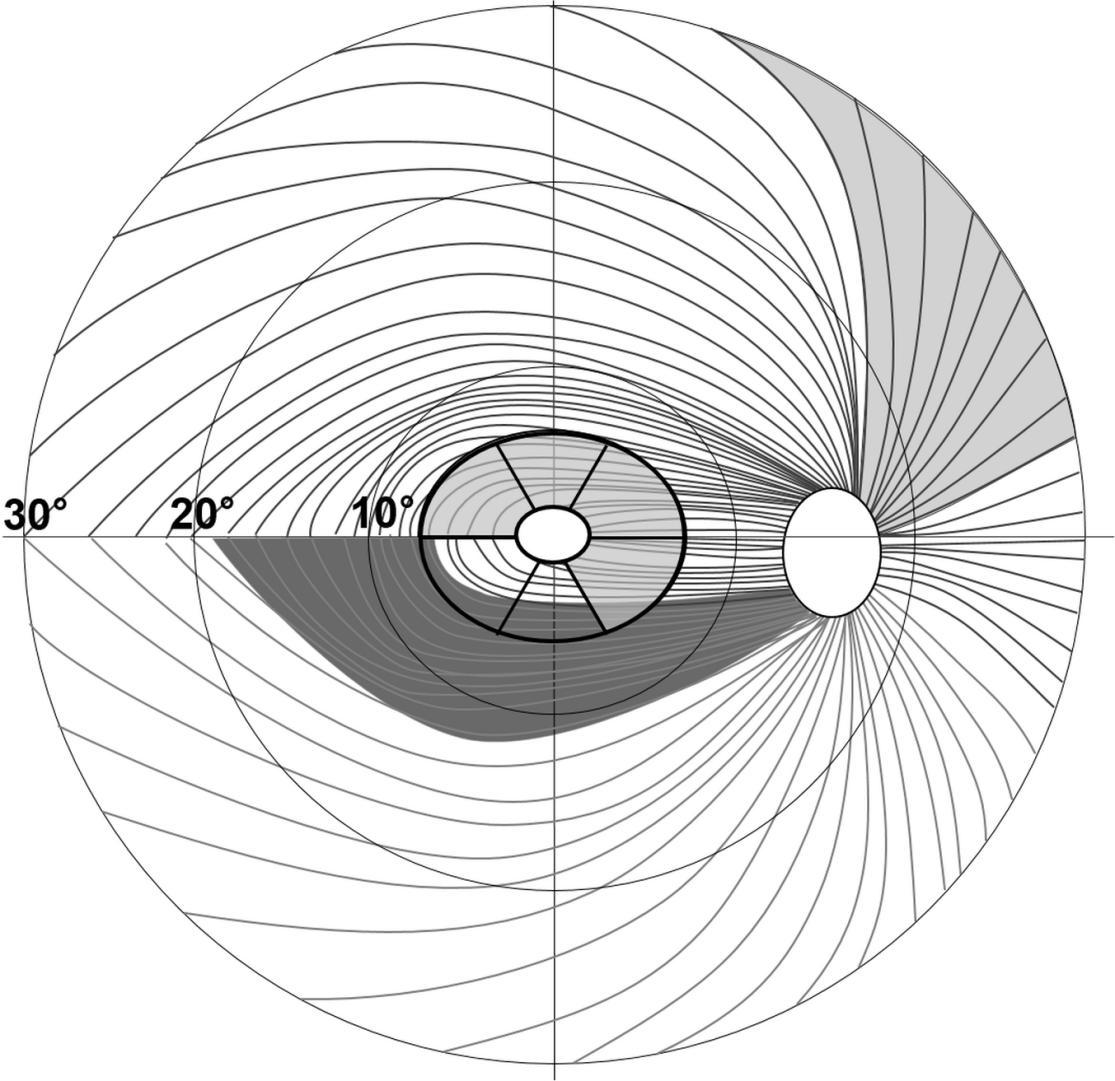
Pattern of the retinal nerve fibre layer (RNFL) and sector map of the macular ganglion cell plus inner plexiform layer (GCIPL) in the right eye. The central elliptical annulus is divided into six sectors. The light grey areas, RNFL at the 1 and 2 o’clock (superonasal) and GCIPL at the superotemporal, superior, superonasal, and inferonasal sectors were significantly more damaged in non-arteritic anterior ischaemic optic neuropathy (NAION) than in primary open-angle glaucoma (POAG). The dark grey area, RNFL at 7 o’clock (inferotemporal), was significantly more damaged in POAG than in NAION.

Table 3 shows comparisons of the axial length adjusted GCIPL thickness parameters between the NAION and POAG groups. Average GCIPL thicknesses of the NAION and POAG were 59.6 ± 16.162 and 64.8 ± 10.5 μm, respectively; there was no significant difference (p = 0.227). However, most of the sectoral GCIPL thicknesses, in the superotemporal, superior, superonasal, and inferonasal sectors, were significantly thinner in NAION than POAG (56.0 ± 14.6 vs. 63.7 ± 11.2 μm, *p* = 0.006; 58.9 ± 13.3 vs. 66.9 ± 12.5 μm, *p* = 0.005; 58.5 ± 16.1 vs. 72.1 ± 11.2 μm, *p* < 0.001; and 60.5 ± 15.8 vs. 67.0 ± 11.2 μm, *p* = 0.003, respectively). Other inferior and inferotemporal GCIPL thickness showed no difference. A schematic sketch of these comparisons of RNFL and GCIPL between NAION and POAG is shown in Fig 1.

**Table 3:** Comparison of the macular ganglion cell plus inner plexiform layer thickness parameters between non-arteritic anterior ischaemic optic neuropathy and primary open-angle glaucoma.

	NAION	POAG	*P* value*
Average	59.6 ± 16.2	64.8 ± 10.5	0.227
Superotemporal	56.0 ± 14.6	63.7 ± 11.2	0.006
Superior	58.9 ± 13.3	66.9 ± 12.5	0.005
Superonasal	58.5 ± 16.1	72.1 ± 11.2	<0.001
Inferonasal	60.5 ± 15.8	67.0 ± 11.2	0.003
Inferior	60.5 ± 15.6	59.8 ± 10.6	0.906
Inferotemporal	62.4 ± 17.2	59.4 ± 12.3	0.590

(μm, mean ± SD)

NAION = non-arteritic anterior ischaemic optic neuropathy, POAG = primary open-angle glaucoma

* Axial length adjusted analysis of covariance.

**Fig 2:**
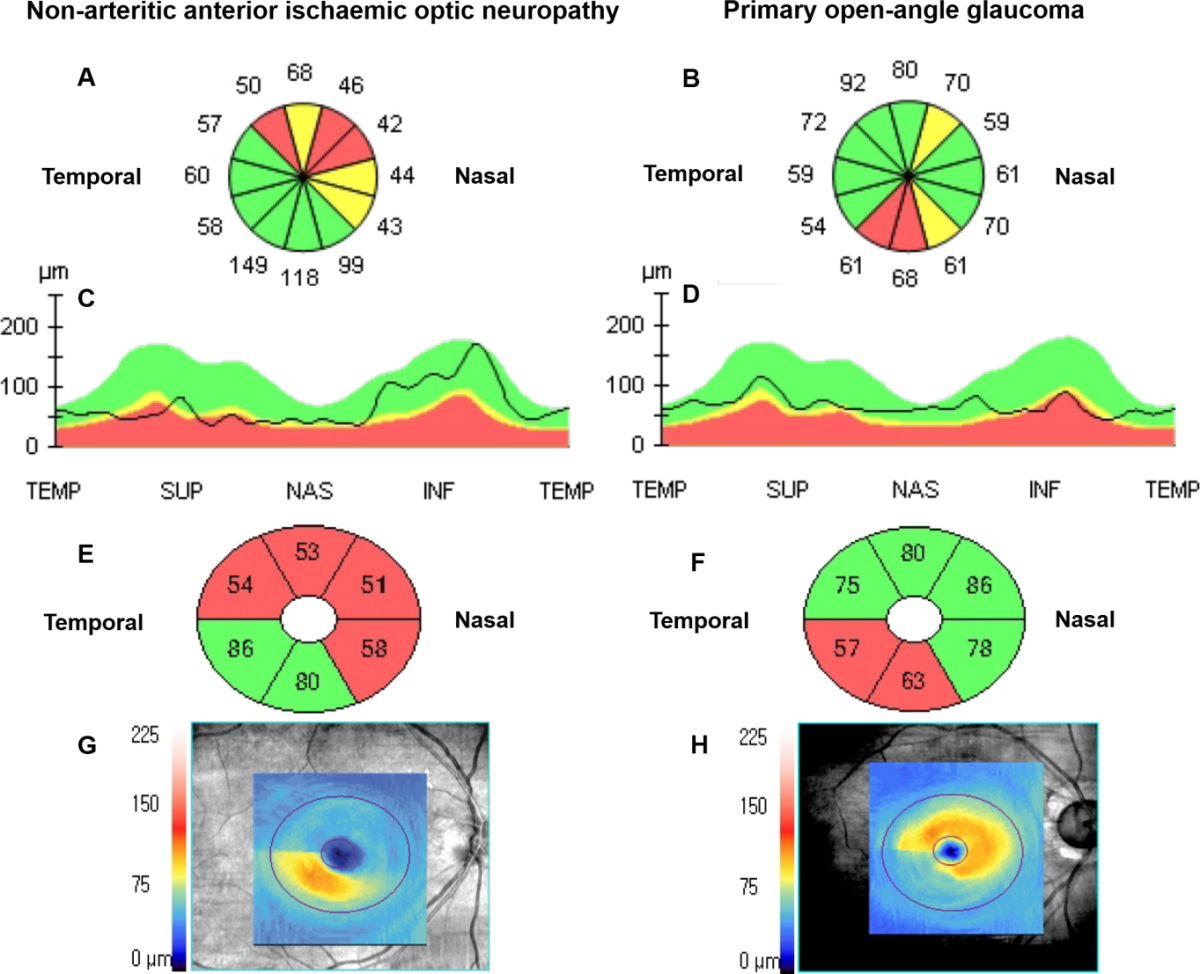
**Optical coherence tomography analysis reports of a representative case of NAION (A, C, E, G) and an age- and average RNFL thickness-matched case of POAG (B, D, F, H).** Average RNFL thicknesses of the NAION and POAG were 69 and 67 μm, respectively. (A, B) Clock-hour RNFL thickness map and (C, D) RNFL thickness profile (TEMP: temporal, 9 o’clock; SUP: superior, 12 o’clock; NAS: nasal, 3 o’clock; and INF: inferior, 6 o’clock) show that significant thinning (coloured red or yellow) is distributed in the superior and nasal regions in NAION, and is located in the inferior region in POAG. (E, F) Sectoral macular GCIPL thickness map and (G, F) GCIPL map show that the significantly thinning area in NAION was larger than that in POAG. Average GCIPL thicknesses in NAION and POAG were 64 and 73 μm, respectively.

OCT printing reports, of the RNFL and GCIPL analyses in representative cases of NAION and age-, sex-, and average RNFL thickness-matched POAG, are shown in Fig 2. The ages of the NAION and POAG cases were 71 and 72 years, respectively, and the average RNFL thicknesses were 69 μm and 67 μm, respectively. According to the clock-hour RNFL thickness map and RNFL thickness profile, the OCT platform rated RNFL defects from borderline to abnormal (yellow and red colour codes, respectively), compared with information in an internal normative database sequentially distributed at the superior and nasal region in NAION. However, RNFL defects were located in the inferior region in POAG. According to GCIPL maps, the average GCIPL thicknesses of the NAION and POAG were 64 μm and 73 μm, respectively, and the OCT platform-rated GCIPL defects from borderline to abnormal (yellow and red colour codes, respectively) in NAION were larger than those in POAG (four vs. two sectors).

### Comparison between NAION and their fellow eyes (Table 4)

The mean difference in the average RNFL thickness between NAION and their fellow eyes was 30.4% (64.7 ± 12.3 vs. 93.0 ± 7.6 μm, p < 0.001). Damaged quadrants exceeding this 30.4% were the superior and inferior quadrants. Damaged clock-hours exceeding 30.4% were from 10 to 1 o’clock and from 5 to 7 o’clock (7 hours).

**Table 4:** Comparison of the peripapillary retinal nerve fibre layer thickness between non-arteritic anterior ischaemic optic neuropathy eyes and normal fellow eyes.

	NAION eye	Fellow eye	Reduction rate (%)*	*P* value†
Average RNFL thickness	64.7 ± 12.3	93.0 ± 7.6	30.4	<0.001
Quadrant RNFL thickness				
Superior	70.8 ± 22.6	111.9 ± 11.8	36.7	<0.001
Nasal	54.4 ± 10.0	67.3 ± 9.8	19.2	<0.001
Inferior	80.0 ± 23.5	124.2 ± 16.1	35.6	<0.001
Temporal	53.1 ± 12.1	68.9 ± 10.2	23.0	<0.001
Clock-hour RNFL thickness				
11 o’clock	73.5 ± 25.2	119.0 ± 22.7	38.2	<0.001
12 o’clock	75.8 ± 27.4	114.0 ± 19.8	33.5	<0.001
1 o’clock	62.5 ± 17.3	103.0 ± 18.1	39.3	<0.001
2 o’clock	57.8 ± 14.8	78.1 ± 16.8	26.1	<0.001
3 o’clock	51.8 ± 9.5	60.3 ± 8.3	14.1	0.005
4 o’clock	53.1 ± 7.8	62.5 ± 10.8	15.0	0.001
5 o’clock	64.4 ± 16.1	98.2 ± 17.3	34.4	<0.001
6 o’clock	81.8 ± 28.9	136.4 ± 25.1	40.0	<0.001
7 o’clock	93.7 ± 29.6	138.2 ± 19.8	44.5	<0.001
8 o’clock	55.1 ± 14.3	69.5 ± 12.2	20.7	0.001
9 o’clock	48.8 ± 10.7	56.0 ± 8.1	13.0	0.004
10 o’clock	55.0 ± 17.7	81.1 ± 15.6	32.2	<0.001

(μm, mean ± SD)

RNFL = retinal nerve fibre layer, GCIPL = ganglion cell plus inner plexiform layer

NAION = non-arteritic anterior ischaemic optic neuropathy

*Reduction rate (%) = 100 × (RNFL thickness of the fellow eye–RNFL thickness of the NAION eye)/RNFL thickness of the fellow eye

† Axial length adjusted linear mixed model.

### Comparison between POAG and their control eyes (Table 5)

**Fig 3:**
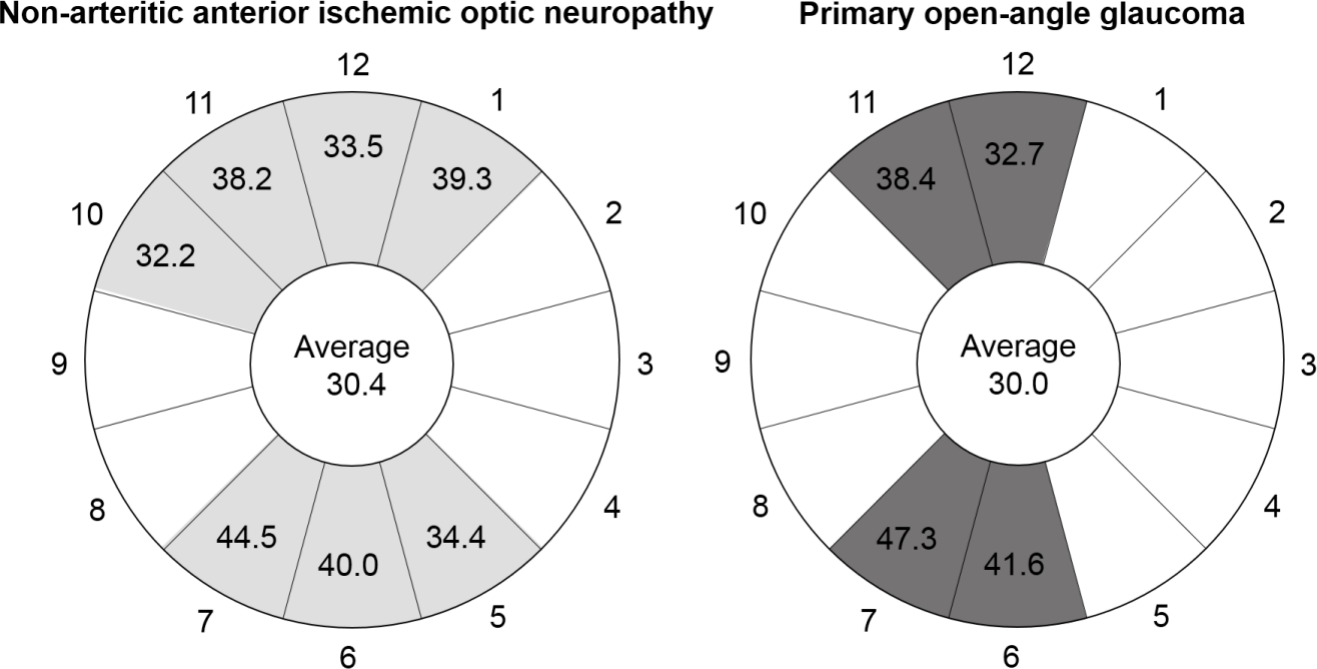
Reduction (%) of clock-hour RNFL thickness in NAION and POAG compared with fellow eyes and normal control eyes, respectively. In both disease groups, the average reductions were 30.4% and 30.0%. Clock-hours with greater than average reductions are in light grey for NAION and dark grey for POAG. 12 o’clock: superior; 3 o’clock: nasal; 6 o’clock: inferior; and 9 o’clock: temporal.

The mean difference in average GCIPL thickness between POAG and their control eyes was 30.0% (64.8 ± 11.7 vs. 92.6 ± 8.2 μm, p <0.001). Damaged quadrants exceeding this 30.0% were the superior and inferior quadrants. Damaged clock-hours exceeding 30.0% were from 11 to 12 o’clock and from 6 to 7 o’clock (4 hours). A schematic sketch of these results is shown in Fig 3.

**Table 5:** Comparison of the peripapillary retinal nerve fibre layer thickness between primary open-angle glaucoma patients and normal controls (μm, mean ± SD).

	POAG	Normal	Reduction rate (%)*	*P* value†
Average RNFL thickness	64.8 ± 11.7	92.6 ± 8.2	30.0	<0.001
Quadrant RNFL thickness				
Superior	77.1 ± 18.9	113.6 ± 13.9	32.1	<0.001
Nasal	58.0 ± 8.8	68.8 ± 10.5	18.6	<0.001
Inferior	71.5 ± 21.8	119.7 ± 13.7	40.2	<0.001
Temporal	53.0 ± 13.4	68.2 ± 11.3	22.3	<0.001
Clock-hour RNFL thickness				
11 o’clock	75.4 ± 23.1	122.3 ± 17.2	38.4	<0.001
12 o’clock	79.6 ± 22.0	118.5 ± 26.2	32.7	<0.001
1 o’clock	76.4 ± 21.7	100.1 ± 17.8	23.7	<0.001
2 o’clock	68.5 ± 15.8	83.9 ± 16.5	18.4	<0.001
3 o’clock	52.2 ± 10.0	58.5 ± 10.6	10.8	<0.001
4 o’clock	53.2 ± 9.1	64.2 ± 11.6	16.8	<0.001
5 o’clock	67.2 ± 14.0	94.9 ± 16.5	29.2	<0.001
6 o’clock	75.2 ± 21.2	128.8 ± 20.5	41.6	<0.001
7 o’clock	72.3 ± 25.4	137.3 ± 16.7	47.3	<0.001
8 o’clock	52.9 ± 13.2	70.4 ± 13.5	24.9	<0.001
9 o’clock	49.3 ± 12.7	54.0 ± 9.6	8.5	0.004
10 o’clock	56.7 ± 17.6	78.7 ± 11.9	28.0	<0.001

RNFL = retinal nerve fibre layer, GCIPL = ganglion cell plus inner plexiform layer

POAG = primary open-angle glaucoma

*Reduction rate (%) = 100 × (RNFL thickness of the normal eye–RNFL thickness of the POAG eye)/RNFL thickness of the normal eye

**†** Axial length adjusted analysis of covariance.

## Discussion

Our results show that there were differences in the more significantly damaged RNFL clock hours between NAION and POAG: RNFL thickness at 7 o’clock was thinner in POAG but RNFL thicknesses at 1 and 2 o’clock were thinner in NAION. Moreover, most of the sectoral GCIPL thicknesses were thinner in NAION, except for the inferior and inferotemporal sectors.

Commonly, glaucomatous optic neuropathy is initiated and most pronounced at the inferotemporal neural rim of the optic disc. [8] It has also been shown that inferior RNFL thickness, among the four quadrant RNFL thicknesses, and the 7 o’clock RNFL thickness, among the 12 clock-hour RNFL thicknesses, have the highest glaucoma diagnostic ability in comparisons of glaucoma patients with normal subjects, according to the areas under receiver operating characteristic curves (AUROC). [34–37] According to our study results, these characteristic features of glaucomatous optic neuropathy, localised optic disc damage seem to be consistently useful for distinguishing it from NAION.

In NAION, the 1 and 2 o’clock (superonasal) RNFL thicknesses were significantly thinner than in POAG, which may be explained in several ways. First, as glaucoma progresses, the nasal neural rim of the optic disc finally becomes thinner. Jonas et al. [38] reported that, in patients with moderate-to-advanced glaucoma, neural rim remnants were usually prominent in the nasal rim, especially the superonasal sector. Additionally, it has been shown that nasal RNFL thickness (around 2, 3, and 4 o’clock RNFL thickness) has the lowest glaucoma diagnostic ability. [34–37] Second, although the resulting altitudinal asymmetry of the optic disc damage, i.e. the difference between superior and inferior, was similar in both diseases, there is a fundamental difference when we consider the mechanism of this optic disc damage. In POAG, altitudinal asymmetry is consistent with the characteristic distribution of retinal nerve fibres [20, 21]. On the other hand, in NAION, the vascular circle (the circle of Zinn-Haller) derived from the short posterior ciliary arteries exhibits distinct upper and lower halves, consistent with the altitudinal damage evident in NAION. [22–24] Thus, in an optic disc hemisphere, regardless of whether it is superior or inferior, optic disc damage in NAION would be expected to be more diffuse from the temporal to nasal neural rim. Finally, ischaemic events in NAION occur more frequently in the superior versus inferior region. [29, 39, 40] Horowitz et al. [41] compared RNFL thicknesses between glaucoma and NAION using a time-domain OCT that did not index clock-hour RNFL thicknesses. In their study, they showed the RNFL thickness profile of a typical NAION case. Interestingly, their profile was similar to ours (Fig 2). The RNFL thickness profile from the superotemporal to the nasal region (clockwise through superior) was almost evenly flat. We do not claim that the 1 and 2 o’clock RNFL region is the most damaged area in NAION, but suggest that this area is relatively more damageable in NAION than in POAG.

In the present study, four sectors of the sectoral GCIPL thickness map (superotemporal, superior, superonasal, and inferonasal) were significantly thinner in NAION. This result may largely be explained by the locational characteristics of the papillomacular nerve fibre bundles. The superotemporal, superior, superonasal, and inferonasal sectors contain more papillomacular nerve fibre bundles that form the temporal neural rim of the optic disc (Fig 1). [42] While papillomacular nerve fibre bundles are spared in this region until a late stage in POAG, they are generally damaged in NAION. [13, 42, 43] The higher incidence of ischaemic events in the superior region in NAION, and less profound damage in the superior region in POAG, may also support this. [29, 34–37, 39, 40] However, in our study, there was no difference in the inferior or inferotemporal GCIPL sectors between NAION and POAG. These sectors overlap with the inferotemporal RNFL pathway (Fig 1) which is commonly affected earlier, and in a more profound way, in glaucoma. [26, 33–37, 44]

Fard et al. [13] compared sectoral GCIPL thicknesses in moderate-to-severe POAG with NAION using Spectralis OCT. The size of the GCIPL measurement area of an elliptical annulus in Cirrus OCT (2.0 mm vertical and 2.4 mm horizontal radius) was larger than the inner circle in Spectralis OCT (3.0 mm diameter), but smaller than the outer circle (6.0 mm diameter). In their study, the four quadrants of the outer GCIPL did not differ between the two diseases, but three of four inner GCIPL quadrants (i.e. all except the inferior quadrant), were significantly thinner in NAION. The results for the inner GCIPL were similar to ours, although they adjusted the MD to make POAG and NAION more similar in terms of severity. The considerably decreased visual acuity in NAION may affect overestimation of MD and degrade the reliability of visual field tests. [29, 45] Furthermore, Danesh-Meyer et al. [46] showed that, for the same amount of visual field loss, based on MD, POAG eyes had a greater reduction in mean RNFL thickness than did AION eyes. That is, MD was worse at the same RNFL thickness for NAION. We consider that this may result from not only greater retinal ganglion cell loss in NAION but also the limitations of the functional test. Thus, we used average RNFL thickness instead of MD as a more objective and reproducible matching index in both disease groups. [30, 31]

We also analysed RNFL thickness reduction in NAION and POAG versus their fellow and control eyes: the degree of average RNFL thickness reduction was similar, at ~30%. However, in damaged clock-hours, above average RNFL thickness reductions were greater in NAION (seven clock-hours vs. four in POAG). This RNFL damage pattern may support our suggestion that optic disc damage in NAION is more diffuse, not only temporally but also in the nasal region, than that in POAG.

Our study was limited by its small sample size. Larger studies are needed to confirm our findings. Second, in almost all NAION cases in this study, the superior region of the optic disc region was mainly affected. Only three patients seemed to be mainly affected in the inferior region (data not shown), Thus, our study result is not representative of patients with inferior NAION. Simply, inferior-affected NAION cases may show more inferonasal RNFL damage than cases with glaucoma. However, according to Aggarwal et al. [10], RNFL damage patterns in NAION are complex. In mainly superior-field-defect NAION (i.e. inferior optic disc affected), sparing of losses in the inferonasal RNFL, and inferior field loss cases showing inferonasal RNFL loss, were common. Third, we enrolled NAION patients at least 3 months after the acute phase (range, 4.5–20.5 months). Although Bellusci et al. suggested that optic disc swelling in the acute phase should have improved by 3 months, another report indicated that RNFL thinning was incomplete until 6 months. Therefore, although the differences in measured thicknesses are likely to be small, some additional RNFL thinning in our NAION sample may not have been taken into account.

In conclusion, at the same average RNFL thickness, the more-damaged o’clock RNFL thickness regions differed between NAION and POAG. In the main, the sectoral GCIPL thickness was thinner in NAION than in POAG. Damaged RNFL clock hours beyond the average RNFL thickness reduction, versus each control, were larger in NAION than in POAG. Thus, the relatively wide extent of RNFL clock-hours damage, especially in the superonasal and/or inferonasal area, and more severe GCIPL damage, in NAION than POAG at the same RNFL thickness, might be helpful in objective differentiation of these two diseases.
